# Can intrinsic loop energetics predict G-Quadruplex topology?

**DOI:** 10.1371/journal.pcbi.1014542

**Published:** 2026-08-18

**Authors:** Michał Jurkowski, Mateusz Kogut, Mikołaj Ławicki, Jacek Czub

**Affiliations:** 1 Department of Physical Chemistry, Gdańsk University of Technology, Gdańsk, Poland; 2 BioTechMed Center, Gdańsk University of Technology, Gdańsk, Poland; Max Planck Institute of Molecular Plant Physiology: Max-Planck-Institut fur molekulare Pflanzenphysiologie, GERMANY

## Abstract

Naturally occurring DNA G-quadruplexes (G4s) regulate many cellular processes, such as gene expression and replication, whereas designed G4s serve as building blocks for controllable nanodevices. Composed of four G-tracts and three loops, G4s exhibit high structural polymorphism, mainly associated with different geometries adopted by the loops. Understanding the biological role of G4s and facilitating their targeted design require detailed knowledge of their folding preferences, which likely originate from the stability of individual loops. However, sequence-dependent preferences for loop geometries and their effects on the overall G4 conformational landscape remain unclear. Here, we used molecular dynamics simulations to systematically evaluate folding free energies of all five standard G4 loop geometries across four loop lengths. Our results reveal that loop-geometry preferences strongly depend on the loop spatial span and, with increasing length, shift from low-span toward large-span geometries. Crucially, we discovered that the overall G4 fold is primarily dictated by internal loop stabilities in three-tetrad G4s but not in two-tetrad ones, where inter-loop interactions emerge as vital stability determinants. Moreover, internal loop stabilities explain the exceptional structural diversity of G4s with three-nucleotide loops reported by experimental studies. Finally, we show that loop-geometry preferences arise from an interplay between the electrostatic repulsion of phosphate groups and loop overstretching.

## Author summary

DNA G-quadruplexes (G4s) are non-canonical DNA structures that play important roles in gene regulation and are increasingly used in nanotechnology. Despite their importance, predicting the exact G4 structure for a given DNA sequence remains challenging due to the high heterogeneity of G4 folded forms. A key source of this structural variability lies in the loops that connect the guanine-rich segments of the structure. In this study, we used molecular dynamics simulations to systematically analyze how the geometry and length of these loops influence G4 folding preferences. We found that loop geometry depends strongly on how far the loop must span: shorter loops favor compact conformations, while longer loops increasingly adopt more extended arrangements. Importantly, we show that the stability of individual loops plays a dominant role in determining the overall structure of G4s with three stacked guanine layers, whereas in simpler two-layer G4s, interactions between loops become more critical. Our results also provide a mechanistic explanation for why G4s with three-nucleotide loops exhibit particularly high structural diversity, as observed experimentally. Overall, this work clarifies the physical principles governing G4 folding and highlights how subtle sequence-dependent effects shape their structural landscape. These insights will help improve predictions of G4 formation in genomes and guide the rational design of G4-based nanodevices.

## Introduction

DNA sequences enriched in guanine tracts (G-tracts) tend to fold into compact secondary structures called G-quadruplexes (G4s), where guanine bases associate together to form a stack of planar tetrads known as the guanine core (G-core), stabilized by Hoogsteen hydrogen bonds and centrally placed metal ions [1–3]. G-quadruplexes are abundant in the human genome. Recent genome-wide ChIP-Seq assay studies revealed their presence in 70 % of genes and 60 % of promoters, with the total number of G4 structures identified in the cell reaching 120,000 [4]. However, as indicated by bioinformatic analysis, the number of sequences potentially capable of forming G4s is even higher, ranging up to 700,000 [5]. Apart from genes and promoters, G4 structures are also present in telomeres, immunoglobulin switch regions, and replication origins, where they are integral to chromosome stability, transcriptional regulation, and replication control [6–17].

In addition to naturally occurring G4s, these structures have also found applications in broadly defined nanotechnology as versatile building blocks for switchable DNAzymes [18,19], conductive molecular wires (G-wires) [20,21], shape-memory materials [22,23], DNA origami structures [24, 25], and controllable drug lock/release systems [26,27].

Due to the many possible ways of assembling a G-core from guanine-rich DNA strands, G4s are topologically diverse structures. This diversity, arising from different orientations of G-tracts and their positioning within the G4 stem, is mainly manifested by the distinct shapes of the loops formed by the sequences separating guanine tracts [28–30]. G4 structures typically contain three loops that define the topology, i.e., its overall folded form, and are the principal determinants of their relative stability [31]. The preference for a given topology strongly depends on the length and nucleotide content of these loop regions in a manner that is not yet fully understood. Understanding this relationship is crucial for elucidating the biological function of naturally occurring G4s, as well as for the rational design of G4s with properties tailored for specific nanotechnological applications.

**Fig 1:**
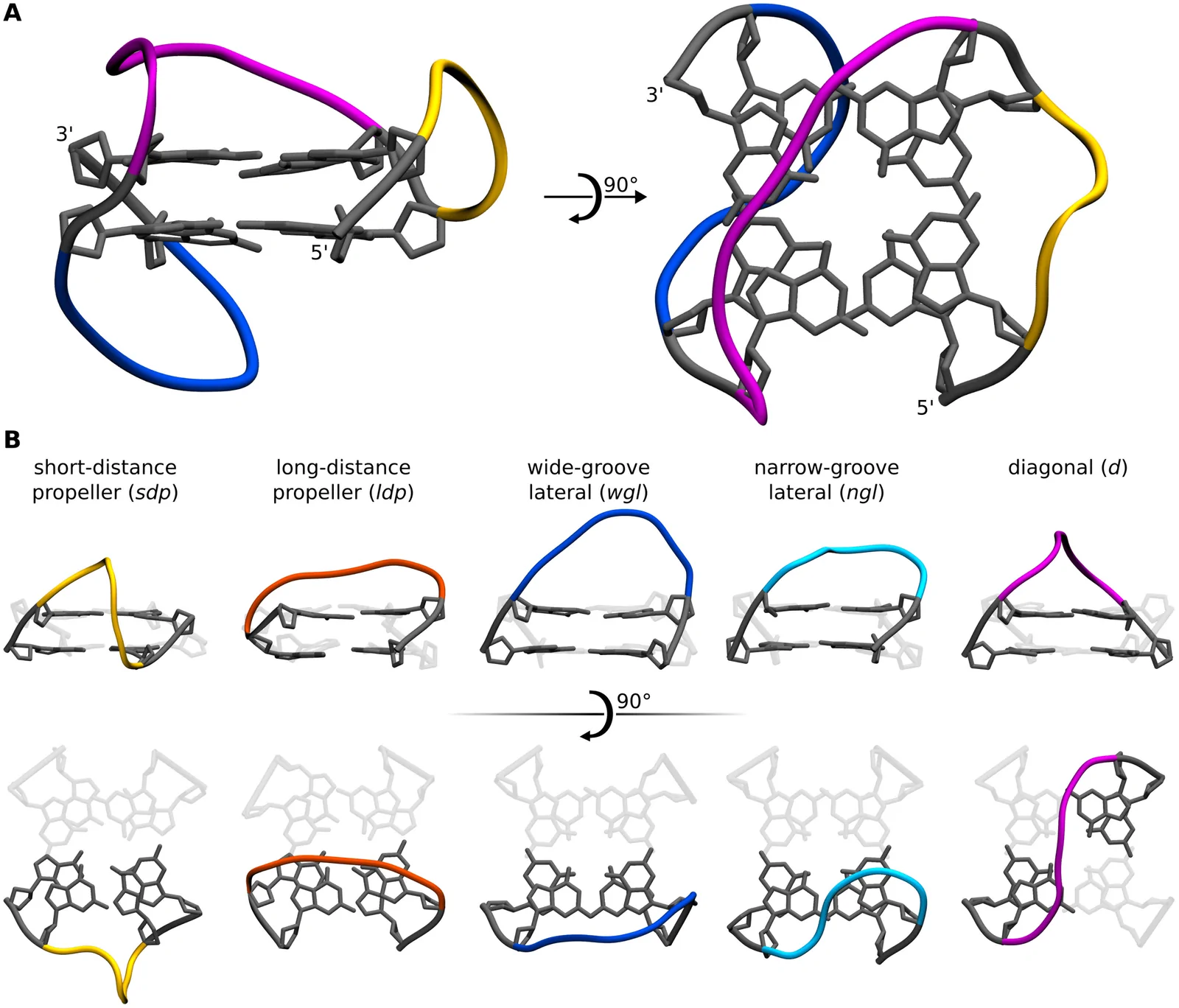
Exemplary G4 structure and depiction of all 5 G4 loop geometries. A: Exemplary G-quadruplex structure featuring short-distance propeller (yellow), diagonal (magenta) and wide-groove lateral (blue) loops of length two, four and three nucleotides respectively. B: Depiction of all five loop geometries found across 26 standard G4 topologies. All loops shown, have the same length of three nucleotides. G-tracts connected by each loop are shown in solid gray, while the rest of the G-core was made transparent for clarity.

Within the 26 standard G4 topologies recognized by Webba de Silva [29], five different loop geometries can be distinguished: short-distance propeller, long-distance propeller, narrow-groove lateral, wide-groove lateral, and diagonal (Fig 1). Short- and long-distance propeller loops both bridge adjacent G-tracts, connecting guanines from the top and bottom tetrads; in typical right-handed G4s, however, the short-distance type attaches to the proximal end of the tilted G-tract, whereas the long-distance type attaches to the distal end. This leads to a substantial difference in the distance that the two propeller loop types must span. Narrow- and wide-groove lateral loops, similarly to propellers, connect adjacent G-tracts; however, unlike propellers, they are attached to guanosines within the same tetrad. The distinction between those two loops is associated with the width of the groove formed by connected G-tracts, again resulting in a different distance spanned by the loop. Finally, a diagonal loop stretches between diagonally opposite G-tracts, connecting guanosines within the same tetrad.

Because each G4 topology represents a specific combination of three loop geometries, it is reasonable to assume that its overall stability arises, at least in part, from the intrinsic stability of these individual geometries. Experimental studies have shown that loop length, loop arrangement, loop residues, flanking sequences, and cation conditions can strongly modulate G4 stability and topology, including cases of topology switching [32–37]. Molecular dynamics simulations have further shown that propeller, lateral, and diagonal loops can sample distinct local conformational ensembles, with loop dynamics coupled to stacking, hydrogen bonding, backbone geometry, and the surrounding G-core [38–40]. However, most previous studies considered complete G4 folds, where the energetic preference of a given loop region for adopting a particular geometry is difficult to separate from the geometry of the remaining loops and from inter-loop interactions. Thus, the intrinsic conformational preferences of individual loop geometries, and their dependence on loop length and composition, remain poorly characterized. It also remains unclear to what extent the overall G4 topology can be predicted from the combined tendencies of its constituent loops.

Despite these challenges, several broad conclusions have been drawn over the years regarding the sequence-dependent conformational preferences of G4 loops. Based on available high-resolution G-quadruplex structures, several empirical relationships between loop length and geometry can be formulated: i) 1-nt-long loops strongly prefer short propeller geometries; ii) narrow and wide lateral geometries generally require loops of at least two nucleotides, with only a few known cases of 1-nt narrow lateral loops [41,42]; and iii) diagonal loops require at least three nucleotides [31,32,43]. Notably, a long-distance propeller loop has never been observed. However, in one G4 structure with a non-standard topology, a 5-nt snap-back loop adopts a conformation resembling that geometry [44]. Beyond these loop-length-dependent constraints, it has been shown that extending a short propeller loop from one to two or three nucleotides decreases the overall stability of the G4 structure, indicating that the intrinsic stability of short propeller loops diminishes with increasing length [33,45]. At the same time, longer loops tend to adopt lateral or diagonal conformations. It remains unclear, however, whether this trend arises from the increased stability of lateral and diagonal loops at greater lengths, or from the destabilization of short-distance propeller loops. To clarify the length-dependent energetic preferences of loop geometries and how they shape the global preferences for G4 topologies, a systematic analysis of the relative stabilities of isolated loops is required.

In this work, we performed a systematic and quantitative analysis of the intrinsic conformational preferences of all major G-quadruplex loop geometries as a function of loop length, aiming to clarify how these preferences shape the stability and topology of G4 folds. By computing loop-folding free energies for isolated loops embedded in two- and three-tetrad G4 scaffolds, we dissected the length-dependent stability differences among loop geometries and linked them to geometry-specific separations of loop attachment points within the G-core. We then combined the resulting additive loop-folding free energies to predict preferred topologies for representative G4-forming sequences and compared these predictions with experimentally determined structures, thereby assessing the extent to which the overall G4 topology can be rationalized from intrinsic loop energetics alone. This approach allowed us to distinguish regimes in which G4 topology is primarily governed by individual loop preferences from those in which additional inter-loop interactions dominate, providing a mechanistic framework for understanding loop-mediated topology selection in G-quadruplexes.

## Methods

### Folding procedure

To determine the folding free energy of individual loops, we analyzed five loop geometries (*sdp*, *ldp*, *ngl*, *wgl*, *d*) across four loop lengths (1–4 nt). For each geometry-length combination, we selected two-tetrad G4 structures in which the first loop (counted from the 5’-end) exhibited the target geometry and length. These starting structures were selected from our *de novo*-folded set comprising all possible two- and three-tetrad G4 conformations with loops containing up to four thymines [46].

Experimental observations show that propeller loops predominantly connect G-tracts adopting *anti-anti* glycosidic bond angles, whereas lateral and diagonal loops most often connect G-tracts in *syn-anti* conformations [35]. Consistent with these preferences, propeller-loop systems were therefore selected with *anti-anti* glycosidic conformations, while lateral and diagonal loop systems were restricted to *syn–anti* conformations.

**Fig 2:**
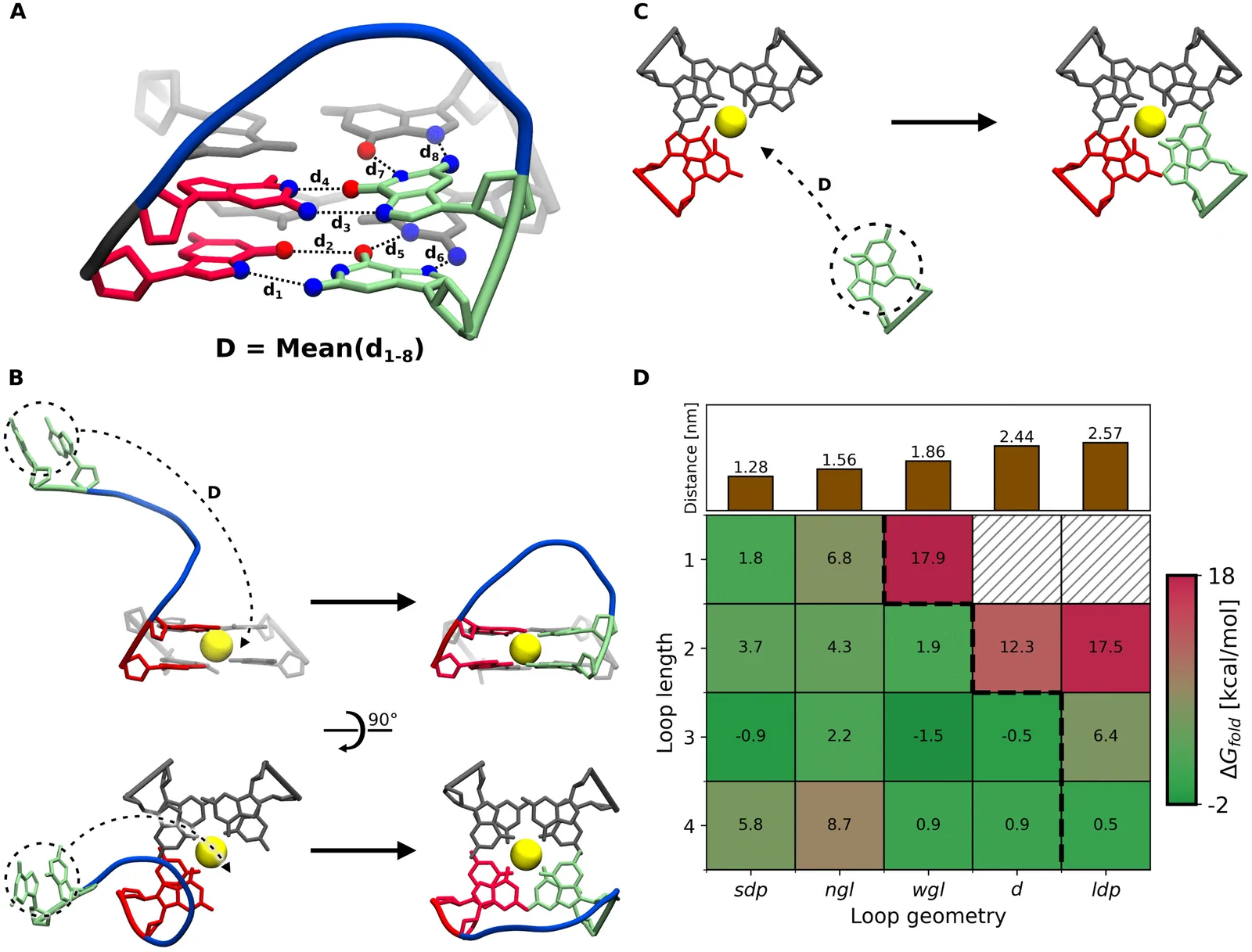
Loop-folding free energies ($\Delta G_{fold}$) for two-tetrad G4s. A: Definition of the reaction coordinate, *D*, used to describe the loop folding process. *D* is defined as the mean pairwise distance between hydrogen-bond donors and acceptors of the 3^′^-end G-tract and those of the two neighboring G-tracts in the preassembled G-core. Donor and acceptor atoms are shown as blue (nitrogen) and red (oxygen) spheres, respectively, and the corresponding pairwise distances are indicated by dashed lines labeled *d*_1_ to *d*_8_. B: Schematic depiction of loop formation from the unfolded state (on the left) to the folded state (on the right). 5’-end G-tract (red) is incorporated into partially formed G-core involving two additional G-tracts in grey. Loop formation in driven by placing 3’-end G-tract (green) in an unoccupied position in the preassembled G-core. C: Schematic depiction of the unconnected G-tract (green) binding to the preassembled G-core (grey). D: Heatmap of loop-folding free energies ($\Delta G_{fold}$) for five loop geometries (x-axis) and four loop lengths (y-axis) in two-tetrad G4s. Hatched entries indicate loop geometries for which $\Delta G_{add}$ could not be determined. The thick dashed line separates loop geometries observed in experimental G4 structures (left) from those not experimentally confirmed (right). Bar plots above the heatmaps show attachment-point separations, defined as shortest surface path, for each loop geometry (see S10 Fig and section B in S1 Text).

To obtain single loop systems, the second and third loops were removed from each selected G4 structure. The resulting strand termini were capped by adding hydrogen atoms to the O5’ and O3’ atoms of the G-tracts. This procedure yielded single-loop systems consisting of a fully-assembled two-tetrad G-core, formed by two G-tracts connected by the loop of interest and two additional unconnected G-tracts (see Fig 2B). A complete list of the single-loop systems analyzed, together with the corresponding parent G4 conformations (including topology and glycosidic bond angles), is provided in S1 Table of the Supporting Information (SI).

To calculate the free energy of an unconnected G-tract binding to the preassembled G-core, we prepared two-tetrad G-core structures without loops (see Fig 2C). Two glycosidic bond angle conformations were considered: *anti–anti* and *syn–anti*. The *anti–anti* G-core was derived from a single-loop structure containing a 1-nt short-distance propeller loop by removing the loop and capping the O5’ and O3’ atoms with hydrogen. Similarly, the *syn–anti* G-core was extracted from a single-loop structure containing a 2-nt wide-groove lateral loop.

**Fig 3:**
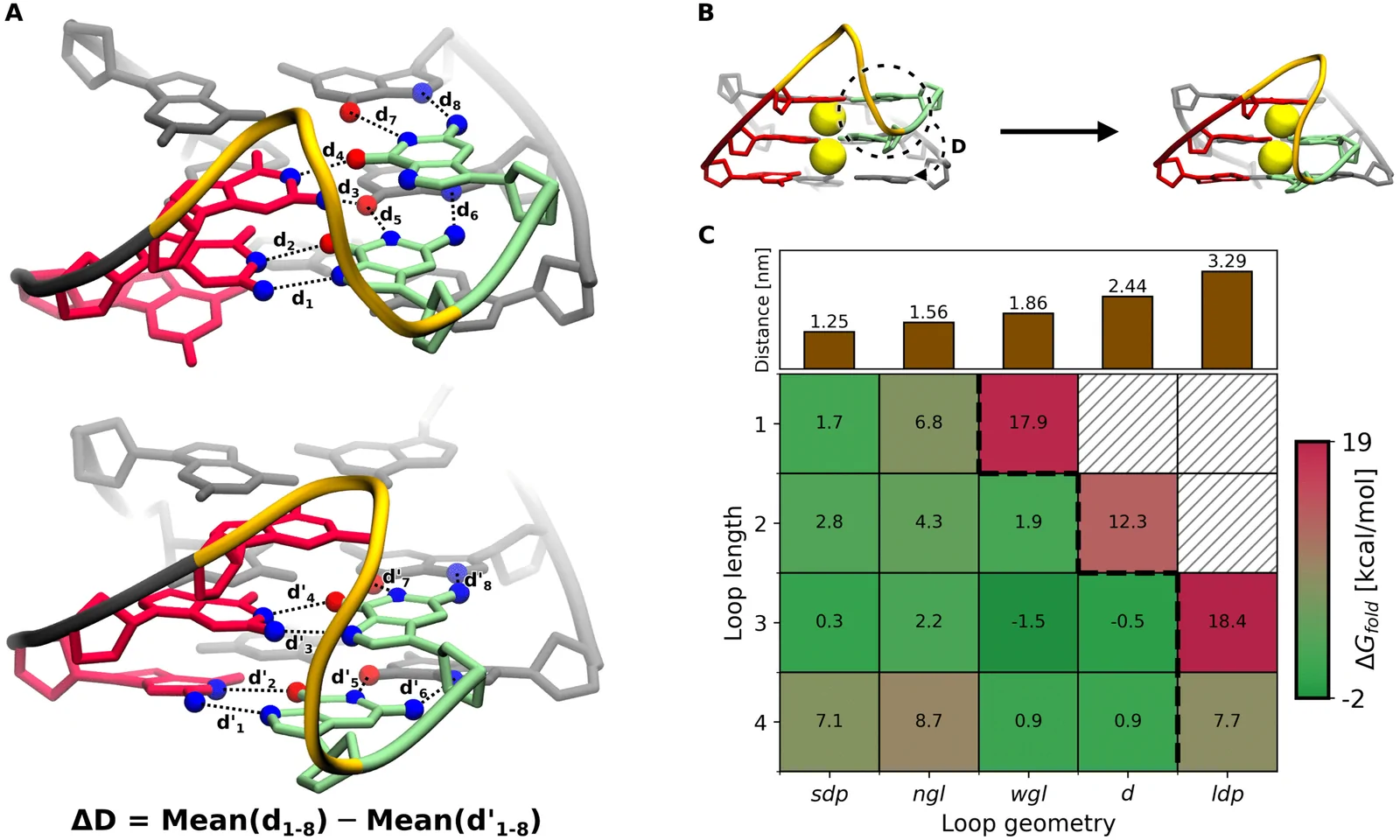
Loop-folding free energies ($\Delta G_{fold}$) for three-tetrad G4s. A: Definition of the reaction coordinate ΔD used to describe the transition of a propeller loop between its two-tetrad-spanning (upper) and three-tetrad-spanning (lower) conformations. ΔD is defined as the difference between the mean pairwise donor-acceptor distances at the top and bottom positions of the 3^′^-end two-guanine tract. These positions correspond to the two-tetrad- and three-tetrad-spanning conformations, respectively. Individual donor–acceptor distances are shown as dashed lines and labeled $d_{1-8}$ (top position) and $d_{1-8}^{\prime }$ (bottom position). B: Shift of the two-guanine G-tract (green) from its upper to lower position within the three-tetrad G-core scaffold, corresponding to propeller’s loop conformational transition from its two-tetrad-spanning to the three-tetrad-spanning conformation, respectively. C: Heatmap of loop-folding free energies ($\Delta G_{fold}$) for five loop geometries (x-axis) and four loop lengths (y-axis) in three-tetrad G4s. Hatched entries indicate loop geometries for which $\Delta G_{add}$ could not be determined. The thick dashed line separates loop geometries observed in experimental G4 structures (left) from those not experimentally confirmed (right). Bar plots above the heatmaps show attachment-point separations, defined as shortest surface path, for each loop geometry (see S11 Fig and section B in S1 Text).

To compute the free energy of the transition between two-tetrad- and three-tetrad-spanning conformations for propeller loops, we selected three-tetrad G4 structures containing the first loop with short- or long-distance propeller geometry and lengths from 1 to 4 nucleotides or 3 and 4 nucleotides, respectively, for these two geometries. The two remaining loops were removed, and the O5’ and O3’ atoms were capped with hydrogen, producing a three-tetrad single-loop structure composed of two loop-connected and two unconnected G-tracts. In addition, the 3’-end loop-connected G-tract was truncated by removing one guanine residue and capping the O3’ atom with hydrogen. The final single-loop structures, therefore, contained three three-guanine tracts and one two-guanine tract connected via a propeller loop to one of the three-guanine tracts (see Fig 3A and 3B).

All prepared G4 structures were placed in the center of a dodecahedral simulation box. Box sizes were determined based on the minimum distance between any G4 atom and the box edges. In the case of single-loop structures, these distances were set to 1.6, 1.9, 2.2, and 2.5 nm for loop lengths of 1–4 nucleotides, respectively, while for structures without loops, to 1.8 nm. All systems were solvated with TIP3P water, and K^+^ and Cl^-^ ions were added to neutralize the system and achieve a physiological ionic strength of 150 mM. For two- and three-tetrad structures, one and two K^+^ ions, respectively, were positioned within the central channel of the G-core.

### Molecular dynamics protocol

All molecular dynamics (MD) simulations were performed using the AMBER bsc1 force field [47]. To assess the sensitivity of the calculated free energy profiles to the choice of potential energy model, an additional set of REUS simulations was performed with the bsc1-VdW force field [48] for selected systems, namely all five loop geometries with 3-nt loops. Simulations were carried out with GROMACS 2020 [49] in combination with the PLUMED 2.6 plugin [50]. The simulations were conducted in the NPT ensemble at a pressure of 1 bar, maintained using the Parrinello–Rahman barostat [51]. The temperature was set to 300 K and controlled with the velocity-rescaling (v-rescale) thermostat [52]. Periodic boundary conditions were applied in all three dimensions. Long-range electrostatic interactions were treated using the Particle Mesh Ewald (PME) method [53], with a real-space cutoff of 1.2 nm and a Fourier grid spacing of 0.12 nm. The same cutoff of 1.2 nm was applied to Lennard–Jones interactions. All bonds involving hydrogen atoms were constrained using the P-LINCS algorithm [54]. The equations of motion were integrated using the leap-frog algorithm with a time step of 2 fs.

### Free energy calculations for loop folding

To explore loop-length–dependent preferences for different loop geometries, we computed free energy profiles for the folding of isolated loops containing one to four thymine residues into each of the five loop geometries.

To this end, we employed replica exchange umbrella sampling (REUS) [55] along a reaction coordinate defined as the mean pairwise distance between atoms involved in the formation of Hoogsteen hydrogen bonds between the 3’-end loop-connected G-tract and its two neighboring G-tracts in the G-core (see Fig 2A).

For each loop geometry, the reaction coordinate was sampled from 0.3 nm – corresponding to the folded state with all eight Hoogsteen hydrogen bonds between the 3’-end G-tract and the core formed – to 2.0, 2.5, 3.0, or 3.5 nm, corresponding to the unfolded state for loops of length 1–4, respectively. Detailed information on the number of windows used in each REUS simulation, as well as the positions and spring constants of the harmonic biasing potentials applied in each window, is provided in S2 Table. All windows were simulated for 4 μs, with exchange attempts between neighboring replicas performed every 2 ps.

Initial configurations for the REUS windows were generated by subjecting each single-loop structure to two successive nonequilibrium unfolding and folding simulations. In the first simulation, loop unfolding was enforced by applying a moving harmonic potential that increased the value of the reaction coordinate from 0.3 nm to the value corresponding to the unfolded state for the given loop length. This potential had a spring constant of 200 kJ/(mol·nm^2^) and was translated along the reaction coordinate at a rate of 0.008 nm/ns. In the second simulation, initiated from the unfolded state, loop refolding was induced by applying a harmonic potential with a spring constant of 400 kJ/(mol·nm^2^) that decreased the reaction coordinate at a rate of 0.004 nm/ns. Initial configurations for all REUS windows were extracted from this second (folding) simulation.

To preserve the integrity of the preassembled G-core, all REUS and unfolding/folding simulations included restraints on the Hoogsteen hydrogen bonds between the 5’-end loop-connected G-tract and the two unconnected G-tracts. These restraints were implemented using a harmonic potential with a spring constant of 250 kJ/(mol·nm^2^), maintaining eight contacts between the hydrogen bond donors (N1 and N2 atoms) and the corresponding acceptors (O6 and N7 atoms). To retain the K^+^ ion within the central channel of the G-core, a harmonic potential with the same spring constant was applied to restrain the ion at a fixed position defined as the center of mass of the O6 atoms of diagonally opposite G-tracts of the preassembled G-core. In addition, to facilitate 3’-end G-tract binding to the empty site of the preassembled G-core, the relative orientation of guanine bases within the 3’-end G-tract was maintained by keeping the root mean square deviation (RMSD) from the referential positions of the guanine atoms at 0 nm with a harmonic potential with spring constant of 1000 kJ/(mol·nm^2^).

Free energy profiles were reconstructed using the weighted histogram analysis method (WHAM) [56]. Uncertainties in the free energy estimates were obtained via a bootstrap procedure that accounts for autocorrelation in the reaction-coordinate time series within each window. The same protocol was applied to all free energy calculations described below.

For details on loop-folding free energy calculation from the free energy profiles, consult section A in S1 Text.

To assess convergence of the profiles, the free energy profiles were evaluated using progressively longer portions of the simulations, from 400 ns to 4000 ns in 400 ns intervals. See S2–S6 Figs for the corresponding plots, which show the free energy profiles obtained at each simulation time for all loop geometries and lengths.

### Free energy calculations for unconnected G-tract binding

To obtain loop-folding free energies that are independent of favorable contributions from G-tract binding, we computed free energy profiles for the binding of an unconnected G-tract to a preassembled G-core. Because loop-folding free energies were evaluated for two distinct glycosidic bond angle conformations of the 3’-end G-tract: *anti–anti* and *syn–syn*, we calculated G-tract binding free energies separately for each of these conformations.

Free energy profiles were computed using replica exchange umbrella sampling (REUS) along a reaction coordinate defined as the mean pairwise distance between atoms involved in the formation of Hoogsteen hydrogen bonds between the binding G-tract and its two neighboring G-tracts in the G-core. For both glycosidic bond angle variants, the reaction coordinate was sampled from 0.3 nm to 2.3 nm, corresponding to the bound and unbound states, respectively. Details of the positions of the harmonic biasing potentials and their spring constants used in individual REUS windows are provided in S3 Table. All windows were simulated for 2 μs, with exchange attempts between neighboring replicas performed every 2 ps.

Initial configurations for the REUS windows were generated via two successive nonequilibrium simulations. In the first simulation, initiated from loop-absent G-core structures, G-tract dissociation was enforced by applying a moving harmonic potential with a spring constant of 200 kJ/(mol·nm^2^), which increased the reaction coordinate at a rate of 0.008 nm/ns. In the second simulation, initiated from the dissociated state, G-tract rebinding to the G-core was induced by applying a moving harmonic potential with a spring constant of 400 kJ/(mol·nm^2^) that decreased the reaction coordinate at a rate of 0.004 nm/ns. Initial configurations for all REUS windows were extracted from this second simulation.

The integrity of the G-core, position of the K^+^ ion within the central channel and relative orientation of guanine bases in the unconnected G-tract were maintained in the same manner as in the loop-folding free energy calculations described above.

To assess convergence of the profiles, the free energy profiles were evaluated using progressively longer portions of the simulations, from 200 ns to 2000 ns in 200 ns intervals. See S8 Fig for the corresponding convergence plots.

### Free energy calculations for propeller loops shift

To assess the relative stability of propeller loops spanning two versus three tetrads, we computed free energy profiles for the positional shift of a two-guanine G-tract between the ‘upper’ and ‘lower’ binding sites within a three-tetrad G-core scaffold. For short-distance propeller loops, four loop lengths (1–4 nucleotides) were considered, while for long-distance propeller loops, two loop lengths (3 and 4 nucleotides) were examined.

Free energy profiles were determined using REUS along a reaction coordinate defined as the difference between the mean pairwise distances of hydrogen bond–forming atoms of the two-guanine G-tract to the G-core guanine bases in the upper versus lower binding site (see Fig 3A). The reaction coordinate ranged from -0.12 nm, corresponding to the two-tetrad-spanning conformation of the propeller loop (with the two-guanine G-tract bound to the upper site), to 0.12 nm, corresponding to the three-tetrad-spanning conformation (with the two-guanine G-tract bound to the lower site). Details regarding the number of REUS windows, the distribution of harmonic biasing potential centers along the reaction coordinate, and the associated spring constants are provided in S4 Table. All windows were simulated for 2 μs, with exchange attempts between neighboring replicas performed every 2 ps.

Initial configurations spanning the reaction-coordinate range were generated by simulating four successive back-and-forth shifts of the two-guanine G-tract between the upper and lower binding sites within the G-core. These shifts were driven by a moving harmonic potential with a spring constant of 1200 kJ/(mol·nm^2^), translated between the reaction-coordinate extrema at a rate of 0.005 nm/ns.

To prevent disaggregation of the G-core, a harmonic restraint with a spring constant of 250 kJ/(mol·nm^2^) was applied to maintain twelve Hoogsteen hydrogen bonds among the three three-guanine G-tracts. The two K^+^ ions located within the central channel were also restrained: one ion was constrained at the center of mass of the O6 atoms of diagonally opposite guanines in the top and middle tetrads, and the second ion at the corresponding center of mass in the middle and bottom tetrads. These restraints were implemented using harmonic potentials with spring constants of 500 kJ/(mol·nm^2^).

In the case of long-distance propeller loops, additional restraints were applied to prevent artificial interactions between loop residues and the vacancy in the G-core created by truncation of one G-tract. To this end, virtual atoms corresponding to guanine base atoms occupying the vacant site were defined, and a harmonic potential with a spring constant of 5 kJ/(mol·nm^2^) was used to enforce zero contacts between loop residues and these virtual atoms. For short-distance propeller loops, in both two-tetrad- and three-tetrad-spanning conformations, loop residues remained solvent-exposed and distant from the vacancy; therefore, no additional restraints were required.

To assess convergence of the profiles, the free energy profiles were evaluated using progressively longer portions of the simulations, from 200 ns to 2000 ns in 200 ns intervals. See S13 and S14 Figs for the corresponding convergence plots.

## Results and discussion

### G4 loop-geometry preferences strongly depend on the loop length

To determine how loop length influences the preference for specific loop geometries, we calculated the free energies of forming single G4 loops of five geometries: *sdp*, *ldp*, *ngl*, *wgl*, and *d*. For each geometry, we considered loop lengths of 1–4 nt.

The loop-forming strand consisted of a thymine segment of 1–4 nt flanked by two-guanine tracts, giving the general sequence 5’-GGT_1−4_GG-3’. To form each loop in the same G4-like structural environment, the 5’-end G-tract of this strand was incorporated into a partially assembled two-layer G-core together with two additional G-tracts (gray in Fig 2B). This arrangement provided a standardized G4-like scaffold and left one binding site empty for the second G-tract of the loop-forming strand.

Loop formation was then simulated by pulling the 3’-end G-tract of the loop-forming strand into the empty site of the preassembled core Fig 2B. The target position and orientation of this G-tract determined the loop geometry. Propeller loops were formed by placing the 3’-G-tract adjacent to the 5’-G-tract in a parallel orientation, lateral loops by placing it adjacent in an antiparallel orientation, and diagonal loops by placing it diagonally opposite to the 5’-G-tract in an antiparallel orientation. In all cases, the final folded state contained eight hydrogen bonds between the 3’-G-tract and the preassembled G-core (Fig 2A; Methods).

Free energy profiles for loop formation were obtained using replica exchange umbrella sampling (REUS; see Methods). These profiles (S1 Fig; see also S2–S6 Figs for convergence tests) describe the free-energy changes along the transition from an unfolded state, in which the loop-forming strand is fully extended, to a folded state, in which the 3’-G-tract is bound to the preassembled core through all eight hydrogen bonds.

The resulting total loop-formation free energies were calculated as the free-energy difference between the folded and unfolded states (see section A in S1 Text for details). These values include two contributions: the intrinsic energetic cost of deforming the thymine segment into the final G4 loop geometry, and the favorable binding free energy gained when the 3’-G-tract binds to the partially assembled G-core. To isolate the loop-folding free energy ($\Delta G_{fold}$) associated with the intrinsic loop-shaping cost, we independently calculated the binding free energies of unconnected G-tracts in the same binding sites (Figs 2C and S7) and subtracted these contributions from the total loop-formation free energies (see section A in S1 Text for details). Because the 3’-G-tract adopted either an *anti-anti* or *syn-anti* glycosidic-bond conformation depending on the loop geometry, the corresponding binding free energies were calculated separately for each conformation.

To test the robustness of the free energy profiles, we also repeated REUS simulations for selected systems, specifically all loop geometries with 3-nt loops, using an alternative potential energy model, the bsc1-VdW force field (see Methods for details). This force field has recently been shown to improve the folding free energy landscape of a model G4 structure [48]. Comparison of the free energy profiles obtained with the two force fields revealed highly similar trends (S9 Fig), indicating that the observed geometry-dependent loop preferences are robust with respect to the choice of force field.

Fig 2D presents the loop-folding free energies for all examined geometries and loop lengths ranging from 1 to 4 nt, except for the 1-nt diagonal and 1-nt long-distance propeller loops. These variants were excluded because severe overstretching and the associated energetic penalties prevented complete folding, precluding reliable free energy calculations.

Overall, loop-geometry preferences appear to be directly related to the spatial separation between the two loop-anchoring guanosines, quantified here as the length of the shortest path between their C3’ and C4’ atoms along the surface of the G-core in the absence of loop (see section B in S1 Text and S10 Fig for definitions and Fig 2D for mean values). One-nucleotide (1-nt) loops are long enough to easily adopt the short-distance propeller conformation, which spans the smallest attachment-point separation distance (~1.28 nm) and is by far the most frequently observed loop geometry of this length in high-resolution G-quadruplex structures. Narrow-groove lateral loops require a somewhat longer span (~1.56 nm) and are 5.1 kcal/mol less stable than short-distance propellers; yet they remain structurally accessible and have been found in G4 structures [41, 42]. In contrast, the remaining geometries (wide-groove lateral, diagonal, and long-distance propeller) require substantially larger spans (1.86, 2.44, and 2.57 nm, respectively) and are highly unfavorable for 1-nt loops ($\Delta G_{fold}$ >17.9 kcal/mol), and have not been reported by structural studies.

With increasing loop length, the preferred loop geometries progressively shift toward those with greater separation between their attachment points. This structural shift is driven by the interplay of two factors: (i) the destabilization of loops constrained to short distances, and (ii) the simultaneous stabilization of topologies that bridge wider separations. In the case of short-distance propeller loops, stability generally declines with increasing length, resulting in $\Delta G_{fold}$ of 2.8 and 5.7 kcal/mol for 2-nt and 4-nt loops, respectively. However, the 3-nt loop presents a notable exception to this trend, showing an unexpected stability increase to -0.9 kcal/mol. Reduced stability of short-distance propeller loops is consistent with experimental findings [33,45], though it should be emphasized that this behavior may not apply to very long loops where additional interactions can compensate for the loss of stability [57,58].

Conversely, narrow-groove lateral loops become increasingly favorable as they extend from 1 to 3 nucleotides (reaching 2.1 kcal/mol), only to suffer a sharp stability penalty of 6.2 kcal/mol upon extension to 4-nt. Wide-groove lateral and diagonal geometries also stabilize with increasing loop length, achieving their highest stability at 3-nt ($\Delta G_{fold}$ = -1.5 and -0.5 kcal/mol, respectively) before experiencing only slight destabilization for 4-nt (by 2.4 and 1.3 kcal/mol, respectively). Finally, the long-distance propeller geometry exhibits a monotonic increase in stability across the entire range of loop lengths examined. It surprisingly emerges as the most stable overall geometry for 4-nt loops, with a folding free energy of 0.4 kcal/mol. Although this result supports the overall energetic drive toward geometries with wider attachment-point separation as loop length increases, it appears to diverge from experimental findings, which have not reported long-distance propeller loops at this loop length. We attribute this discrepancy to the fact that our model was designed to characterize individual loops independently and, therefore, does not capture potentially unfavorable interactions between a long-distance propeller and neighboring loops in a complete G-quadruplex scaffold.

Notably, the computed positive free-energy values for nearly all G-quadruplex loop conformations suggest that most are intrinsically energetically disfavored when isolated. With the exception of 3-nt loops, the intervening sequences do not tend to spontaneously adopt G4-like loop geometries and appear to depend on the stabilizing hydrogen bonds formed between the G-tracts.

In summary, loop-geometry preferences shift markedly with loop length, driven by the destabilization of short-distance propellers and the progressive stabilization of lateral and diagonal geometries. Longer loops also enable the long-distance propeller to become energetically competitive—reaching the lowest predicted free energy at 4 nt; however, this geometry is likely suppressed in actual G-quadruplexes by inter-loop interactions that are absent in our single-loop models. The overall positive free energies confirm that isolated loops are generally unstable and, except for 3-nt sequences, rely on the complete G-quadruplex for structural adoption.

### Conformational energetics of individual loops is similar in three- and two-tetrad G4s

Loop-folding free energies shown in Fig 2D were determined for a single-loop G4 structure with two guanine tetrads; however, three-tetrad G4s are generally more stable [59] and, consequently, more relevant in biology and for design purposes. Therefore, we extended our studies to the three-tetrad G4s. Because lateral and diagonal loops start and end within the same G-tetrad, their geometry should be independent of the number of G-tetrads; thus, our results for these loop geometries can be assumed to transfer directly to three-tetrad G4s. On the other hand, propeller loops spanning across G-tetrads may be expected to differ substantially in folding free energy between two- and three-tetrad G4s.

Because the direct calculation of the loop-folding free energy for propellers in three-tetrad G4s required extensive sampling, which yielded unreliable free energy evaluations within reasonable timescales, we adopted an alternative approach. Our strategy was to first assess the intrinsic folding free energy difference between the two-tetrad- and three-tetrad-spanning propeller loop conformations. This difference was found by simulating the two-guanine 3’-end G-tract’s ‘upper’ to ‘lower’ position shift within the three-tetrad G-core scaffold (Fig 3A and 3B). This simulated shift directly models the loop’s two-tetrad- to three-tetrad-spanning conformational change. We then added this calculated free energy difference (see S12 Fig for the free energy profiles and S13 and S14 Figs for the convergence) to the more reliable folding free energies derived from the two-tetrad systems to obtain the final loop-folding free energies for propeller loops in three-tetrad G4s.

Fig 3C shows the loop-folding free energies for three-tetrad G4s, derived from the assumptions for lateral and diagonal loops and the additional calculations for propeller loops described above. For short-distance propellers, the resulting $\Delta G_{fold}$ values differ only slightly from those of two-tetrad G4s, with a maximum deviation of 1.4 kcal/mol across all loop lengths. Although such minor changes might seem unexpected, they agree with the very small increase in attachment-point separation (~0.03 nm; see also S11 Fig), which may allow the loop to preserve essentially the same conformation in both two- and three-tetrad variants. The minimal net change in separation occurs because the increased distance from spanning an extra tetrad is nearly offset by the decrease in distance caused by the more extensive twist of the outer tetrads (see S15 Fig).

In contrast, long-distance propeller loops show pronounced differences in folding free energy between their two-tetrad- and three-tetrad-spanning forms. In three-tetrad G4s, the formation of a long-distance propeller incurs a substantial stability penalty compared to its two-tetrad counterpart: the 3-nt loop is ~12 kcal/mol less stable, and the 4-nt loop is ~7.2 kcal/mol less stable. This penalty likely reflects the overstretching of the loop as it is forced to span three tetrads. As a result, 3-nt loops cannot adopt a long-distance propeller geometry, and the preference of 4-nt loops for this geometry is greatly diminished. The overstretching arises from a considerable increase in attachment-point separation (~0.72 nm; see also S11 Fig), driven by a stronger twist of the outer tetrads that, unlike for short-distance propellers, does not offset the extended span but instead pushes the attachment points farther apart (see S15 Fig).

In brief, loop-folding free energies in three-tetrad G4s differ only marginally from those in two-tetrad G4s, resulting in largely similar geometry preferences. The only exception is the long-distance propeller geometry, which in three-tetrad G4s becomes highly unfavorable due to severe loop overstretching caused by the increased separation of attachment points, even for 3- or 4-nt loops.

### Loop-geometry preferences determine topological landscape in three-tetrad G4s but not in two-tetrad ones

G4 structures typically contain three loops, whose geometries together define the overall topology. Accordingly, the propensity to adopt a given topology can be expected to depend strongly on the geometry preferences of the individual loops. To assess how the conformational energetics of isolated loops shape the topological landscape of G-quadruplexes, we used our loop-folding free energies to predict preferences among all 26 standard G4 topologies for a set of DNA sequences and compared these predictions with the experimentally observed folds. Specifically, we selected G4-forming sequences with topologies confirmed by high-resolution structural data and with loop lengths not exceeding 4 nt. For each sequence, all 26 topologies were ranked using an additive total loop-folding free energy, $\Delta G_{add}$, constructed by summing the three individual loop contributions ($\Delta G_{fold}$) corresponding to the loop geometries present in a given topology (see S16 Fig) and the loop lengths within the sequence. Accordingly, $\Delta G_{add}$ represents the total loop-folding free energy contribution to the overall stability of the topology for a certain sequence. As a result, each topology for a given sequence was assigned a rank between 1, referring to the lowest (most favorable) $\Delta G_{add}$ and 26, referring to the highest (most unfavorable) $\Delta G_{add}$.

Because $\Delta G_{fold}$ values for diagonal and long-distance propeller loops could not be reliably determined at short lengths (as discussed above), we substituted the missing values with those of the shortest available loop of the same geometry, assuming that shorter loops cannot be more stable than the shortest characterized case (S17 Fig). This procedure yields a lower bound for $\Delta G_{fold}$ for these highly unfavorable geometries at short loop lengths.

**Fig 4:**
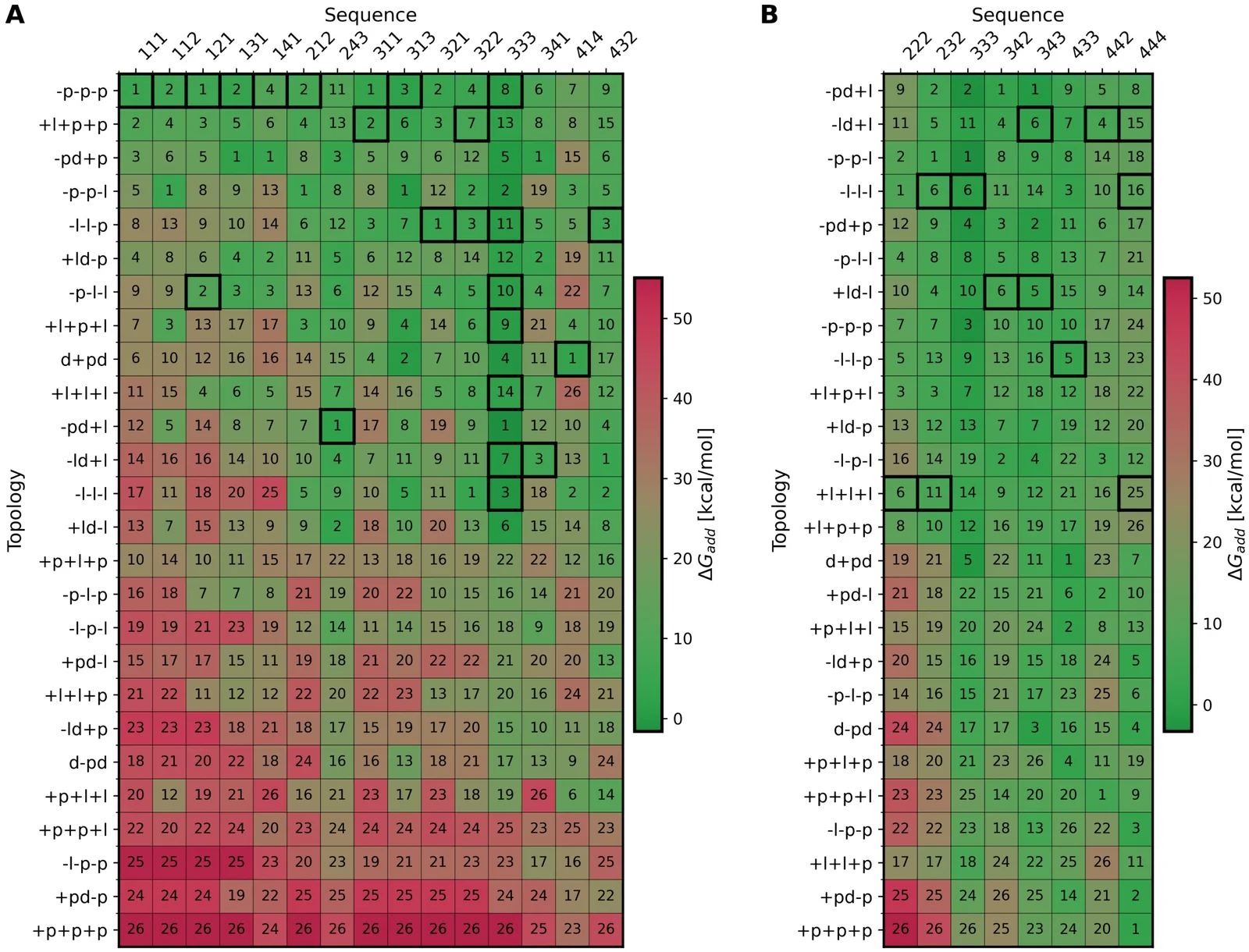
Additive loop-folding free energies ($\Delta G_{add}$) for selected G4 sequences forming two- and three-tetrad G4s. A: Heatmap of additive loop-folding free energies ($\Delta G_{add}$) for 15 sequences forming experimentally observed three-tetrad G4 structures (x-axis), denoted by three-digit codes corresponding to loop lengths, versus the 26 standard G4 topologies (y-axis). Numbers in each cell (1–26) indicate the rank of a given topology for each sequence based on $\Delta G_{add}$, with 1 corresponding to the most favorable (lowest $\Delta G_{add}$) and 26 to the least favorable (highest $\Delta G_{add}$). Experimentally observed topologies for each sequence are highlighted with black frames. B: Analogous heatmap for 8 sequences forming experimentally observed two-tetrad G4 structures. For detailed values of $\Delta G_{add}$ for all considered sequences, inspect S18–S20 Figs.

Fig 4A and 4B summarize the computed $\Delta G_{add}$ values and the corresponding ranks for all 26 topologies across the selected sequences capable of forming three-tetrad and two-tetrad G4s, respectively. Strikingly, for most three-tetrad sequences, the experimentally determined topology or topologies were ranked among the three most stable predictions based on the additive loop-folding free energy. Moreover, for 5 of the 15 sequences considered, the topology with the lowest predicted $\Delta G_{add}$ exactly matched the experimental structure. In only three cases, the experimentally observed topology rank 4th or lower, corresponding to a relatively unfavorable additive free energy. Together, these results indicate that the stability of the G4 topology largely arises from the combined geometry preferences of individual loops, supporting the notion that the topological landscape of three-tetrad G4s is predominantly governed by the intrinsic conformational energetics of their constituent loops.

The largest discrepancy between predicted and experimentally observed topologies was found for the 333 sequence, which exhibits exceptional topological diversity, with seven distinct topologies reported across experimental structures. Of these, only two (−l−l−l and −ld+l) ranked among the seven most stable topologies based on the $\Delta G_{add}$ score (ranks 3 and 7, respectively), whereas the remaining five experimentally observed topologies were assigned ranks between 8 and 14. However, the $\Delta G_{add}$ values obtained for the 333 sequence point to an unusually flat energetic landscape compared to other sequences, with predicted free energies spanning only from -1.60 to 2.87 kcal/mol for topologies ranked up to 14 (see S18 and S19 Figs for detailed values). Such a shallow landscape, arising from the intrinsic conformational energetics of 3-nt loops under the assumption of additivity, provides a natural explanation for the pronounced topological diversity observed experimentally for this sequence. At the same time, it underscores the importance of additional determinants of G4 stability, such as loop base composition, presence of flanking overhangs, cation identity, or molecular crowding, which can readily perturb the delicate balance between topologies defined by loop energetics alone and thereby shift topology preferences [37,60–66].

In contrast to three-tetrad G4s, the ability of $\Delta G_{add}$ to capture topology preferences in two-tetrad G4s is markedly reduced (Fig 4B; see also S20 Fig for detailed values), with experimentally observed topologies spanning a wide range of predicted ranks, from 4 to 25. This outcome is unexpected, given that two-tetrad G4s display loop geometry preferences similar to those observed for three-tetrad G4s (Figs 2D and 3C). The poor correspondence indicates that, in two-tetrad G4s, the topological landscape is governed to a much lesser extent by loop-length-dependent geometry preferences of individual loops. Instead, additional determinants beyond the combined energetics of isolated loops must play a dominant role in shaping topology preferences.

Because two-tetrad G4s are intrinsically less stable than three-tetrad G4s, their formation and topology preferences likely rely on additional stabilization from inter-loop interactions. Notably, all experimentally observed two-tetrad topologies except –l–l–p contain two lateral loops on the same face of the G4, enabling such interactions. In addition, two-tetrad G4 structures are depleted in short-distance propeller loops, which are solvent-exposed and incapable of providing stabilizing inter-loop contacts. These observations indicate that, in two-tetrad G4s, topology selection is driven more by the requirement for inter-loop stabilization than by the intrinsic geometric preferences of individual loops.

In summary, the geometry preferences of individual loops appear to be the primary determinants of topology stability in three-tetrad G-quadruplexes. Notably, the 333 sequence exhibits exceptional topological flexibility, owing to the high similarity of additive loop-folding free energies across a broad range of topologies, consistent with the pronounced topological diversity observed experimentally. In contrast, two-tetrad G4s preferentially adopt topologies that enable additional stabilization through inter-loop interactions, rendering their topological landscape less dependent on the intrinsic conformational energetics of individual loops.

### Loop-geometry selection is governed by an interplay between loop overstretching and repulsion of phosphate groups

**Fig 5:**
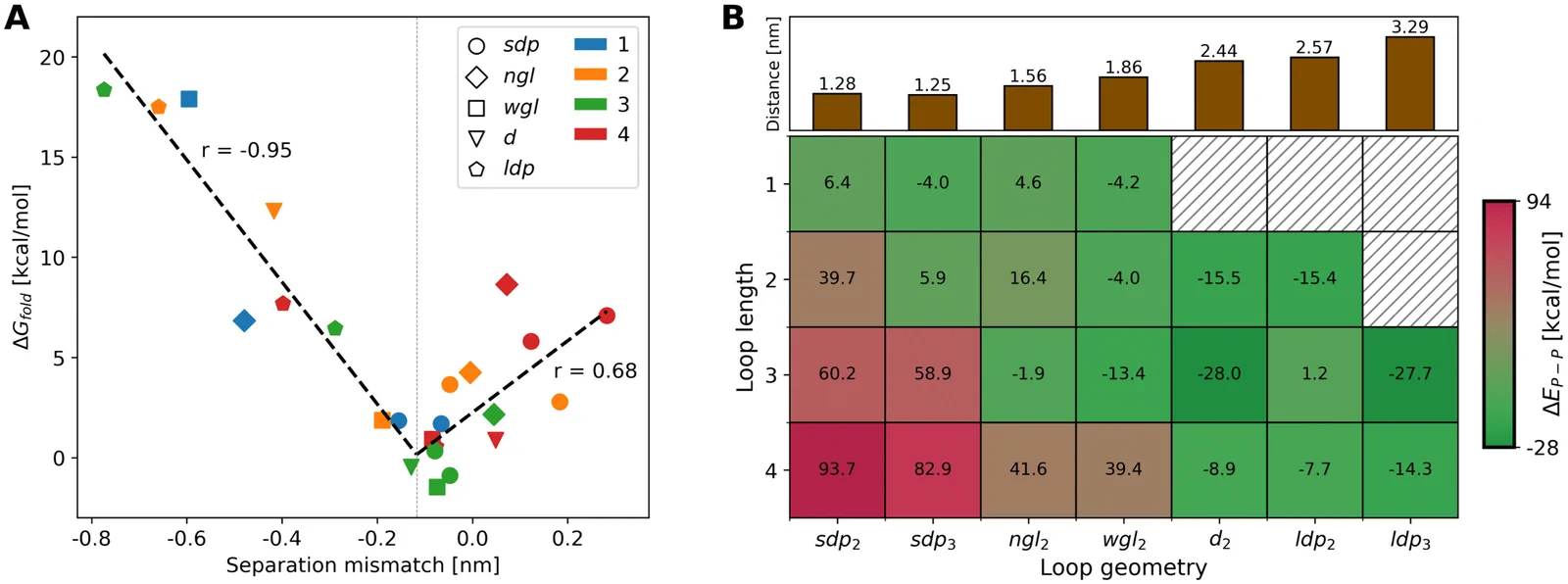
Effect of the attachment-point separation mismatch and repulsion of phosphate groups on the loop-folding free energy. A: Loop-folding free energy as a function of attachment-point separation mismatch (see text for definition and S21 and S22 Figs for exact mismatch values) for all loops considered. Marker shapes and colors denote loop geometry and loop length, respectively. Thick dashed lines show linear models fitted using segmented linear regression, with the breakpoint separating the two mismatch regimes indicated by a thin vertical dashed line. Pearson correlation coefficients (*r*) were calculated separately for each segment. B: Heatmap of the difference in phosphate–phosphate interaction energy between the folded and unfolded states ($\Delta E_{P-P}$) for all loop geometries in two-tetrad G4s and propeller geometries in three-tetrad G4s (x-axis), shown across four loop lengths (y-axis). The number of tetrads for each geometry (2 or 3) is indicated as a subscript in the loop-geometry label. Hatched entries denote loop geometries for which $\Delta E_{P-P}$ could not be determined. The bar plot above the heatmap reports reference attachment-point distances for each loop geometry, calculated as shortest paths along the surface of the loop-absent G-core (see Methods).

As shown in Figs 2D and 3C, loop-folding free energies depend strongly on the geometry-specific separation between loop attachment points, measured as the shortest path along the surface of a fully-assembled G-core in the absence of a loop. This relaxed (reference) separation imposes a spatial constraint that loops of different lengths satisfy with varying degrees of conformational strain, making certain loop lengths better suited to span a given separation distance than others. Consequently, loops whose enforced attachment-point separation closely matches the relaxed reference separation are expected to be more favorable than those that impose either shorter or longer distances. To test whether global loop energetics indeed depend on deviations from this relaxed separation, we plotted loop-folding free energies ($\Delta G_{fold}$) as a function of the separation-distance mismatch, defined as the difference between loop-enforced and reference attachment-point separations (Fig 5A).

As can be seen, at large negative separation mismatches, loops experience pronounced overstretching because the attachment points enforced by the loop are substantially closer than the relaxed separation, resulting in high folding free energies. As the mismatch approaches zero, thereby reducing the extent of unfavorable overstretching, the folding free energy decreases and reaches a minimum at slightly negative mismatch values. Across the entire negative mismatch regime, folding free energy correlates strongly with the separation difference, with a Pearson correlation coefficient (*r*) of –0.95. Strikingly, as the mismatch becomes positive, loop-folding free energies increase again, exhibiting a positive correlation with the separation mismatch (*r* = 0.67). This behavior indicates that unfavorable energetic contributions arise not only from loop overstretching but also from overly short attachment-point separation, leading to loop “squeezing,” likely associated with destabilizing repulsion of phosphate groups.

The observed relationship between the separation mismatch and $\Delta G_{fold}$ provides a natural explanation for the exceptional stability of 3-nt loops. Specifically, all types of 3-nt loops, except for unfavorable long-distance propellers, cluster near zero mismatch values (–0.13 to 0.04 nm), indicating a minimal-strain fit to the reference attachment-point separation. At the same time, the similarity of mismatch values across loop geometries implies that 3-nt loops are comparably well suited to satisfy the spatial constraints of all geometries except for long-distance propellers. This near-universal geometric compatibility likely underlies the remarkable topological versatility observed for the 333 sequences (seven distinct experimentally observed topologies; see Fig 4A).

To test whether loop squeezing leads to destabilization through enhanced electrostatic repulsion of phosphate groups, we computed the difference in the phosphate-phosphate interaction energy between the folded and unfolded states ($\Delta E_{P-P}$) for each loop considered. As shown in Fig 5B, the largest $\Delta E_{P-P}$ values are observed for loop geometries characterized by short attachment-point separations on the G-quadruplex core and generally increase with loop length.

This trend suggests that longer loops must adopt increasingly compressed conformations to satisfy geometry-specific attachment-point separations, bringing phosphate groups into closer proximity. Consequently, this effect is most pronounced for geometries with small attachment-point separations, particularly short-distance propellers, which experience substantial phosphate–phosphate repulsion even for 2- and 3-nt loops. Consistent with the length dependence, the most extreme electrostatic penalties for this geometry are observed for 4-nt loops, with $\Delta E_{P-P}$ reaching approximately 94 and 83 kcal/mol in two- and three-tetrad G4s, respectively, resulting in the pronounced destabilization of these loop conformations.

In contrast, loop geometries with larger attachment-point separations exhibit markedly lower electrostatic repulsion, even for longer loops, as the wider loop span permits a more favorable spatial distribution of phosphate groups.

Together, these results reveal a general trend in which long loops constrained to span short attachment-point separations become increasingly compressed, leading to unfavorable phosphate crowding and consequent electrostatic destabilization. Conversely, loop geometries with larger attachment-point separations favor more extended conformations that alleviate phosphate–phosphate repulsion; however, such geometries cannot be readily adopted by short loops owing to the energetic cost of loop overstretching.

## Conclusion

In this work, we systematically evaluated conformational preferences across all major G-quadruplex (G4) loop types containing one to four nucleotides, with the aim of assessing the extent to which individual loop preferences contribute to the overall stability of G4 folds. Loop-folding free energies calculated separately for two- and three-tetrad G4s reveal that conformational preferences vary markedly with loop length. Short-distance propeller loops, which are strongly favored for 1-nt loops, progressively lose stability as loop length increases, while alternative loop geometries become increasingly favorable and ultimately outcompete short-distance propellers. This shift in geometry preference correlates closely with geometry-specific differences in the separation of loop attachment points within the G-core scaffold, with longer loops preferentially stabilizing geometries characterized by larger attachment-point separations.

To assess the extent to which length-dependent geometry preferences of individual loops shape the topological landscape of G-quadruplexes, we used additive loop-folding free energies to predict preferred topologies for a set of G4-forming sequences and validated these predictions against experimental structures. Crucially, for three-tetrad G4s, the predicted most stable topologies showed strong agreement with the experimentally observed folds. These results indicate that the topological landscape of three-tetrad G4s is largely governed by the intrinsic folding preferences of individual loops.

By contrast, topology preferences in two-tetrad G4s depend far less on intrinsic loop energetics. Instead, these systems preferentially adopt topologies that enable stabilizing inter-loop interactions, even when such topologies are not favored based solely on additive loop-folding energetics.

Interestingly, most computed loop-folding free energies were positive, indicating that loop formation within G4s is generally thermodynamically unfavorable and must be compensated for by stabilizing contributions from guanine-guanine hydrogen bonding within the G-core. Negative folding free energies were observed exclusively for 3-nt loops, highlighting their exceptional intrinsic stability.

Furthermore, the additive folding free energies calculated for the 333 sequence reveal a notably shallow energetic landscape spanning a broad range of topologies, consistent with a strong propensity of this sequence to adopt multiple conformations. This predicted topological versatility aligns well with experimental observations reporting an unprecedented total of seven distinct G4 topologies for this sequence.

Finally, we show that loop geometry preferences are governed by how loops of a given length satisfy the geometry-specific separation of attachment points. Short loops preferentially adopt geometries with small attachment-point separations, as they incur severe energetic penalties from overstretching when forced to span larger separations. Conversely, long loops readily accommodate large separations but must compress to fit geometries with small attachment-point separations, leading to increased phosphate–phosphate repulsion and consequent loop destabilization.

Although the present analysis highlights an important role of loop geometry in G4 topology selection, G4 stability is also shaped by sequence- and context-dependent effects beyond loop length. These include the identity of loop nucleobases, since loop sequence and loop permutation can substantially affect both topology and stability, whereas the present calculations were restricted to thymine loops [64]. Flanking nucleotides may also reshape the conformational ensemble by interacting with G-tetrads and loops or by modulating the *syn*/*anti* preferences of 5’-end guanines [65,67]. More broadly, *syn*/*anti* patterns within the G-core itself contribute to the complexity of G4 folding landscapes [68,69]. Finally, particularly in two-tetrad G4s, inter-loop interactions can render individual loop contributions to overall stability non-additive, introducing stabilizing effects that are not captured by independent loop energetics [35,70]. Future extensions of the present framework could therefore incorporate these effects as additional sequence- and topology-dependent energetic contributions.

In addition to these physical determinants of G4 stability, the quantitative free-energy estimates may also depend to some extent on the chosen force field, although the additional bsc1-VdW calculations performed here support the robustness of the main geometry-dependent trends observed in this work.

Regardless of these limitations, our findings provide a mechanistic framework for understanding loop-mediated topology selection in G-quadruplexes and offer practical guidelines for the rational design of G4 structures for nanotechnological applications. In particular, intrinsic loop-folding preferences can be leveraged to restrict the accessible topological space to the most stable conformations, after which equilibrium can be further biased toward a desired fold through complementary design strategies, including modulation of loop nucleobase composition, incorporation of targeted chemical modifications, addition of flanking sequences, and optimization of environmental conditions.

## Supporting information

S1 Text The following supporting file contains supplementary methods describing the derivation of folding free energies from free energy profiles and the calculation of distances between loop attachment points. (PDF)

S1 Table Summary of single-loop systems. All single-loop systems were extracted from two- and three-tetrad G4 structures obtained via *de novo*-folding [46]. For each single-loop system, the complete characteristic of the parent structure in provided, including the sequence (3-digit codes denoting lengths of thymine loops), topology, and glycosidic bond conformation patterns in 5’-end G-tract of the parent structure.
(PDF)

S2 TableDetails of harmonic potentials employed in each REUS window for loop-folding free energy calculations, for all considered loop geometries (Geom.) and lengths (Len.).r_0_ [nm] specifies the position of the harmonic potential along the reaction coordinate, while κ [kJ/(mol·nm^2^)] indicates the spring constants.(PDF)

S3 TableDetails of harmonic potentials employed in each REUS window for the free energy calculations of the unconnected G-tract binding for *anti-anti* and *syn-anti* glycosidic bond conformations of G-tract.r_0_ [nm] specifies the position of the harmonic potential along the reaction coordinate, while κ [kJ/(mol·nm^2^)] indicates the spring constants.(PDF)

S4 TableDetails of harmonic potentials employed in each REUS window for the free energy calculations of propeller’s loop transition between two-tetrad- and three-tetrad-spanning conformations, for short- and long-distance propeller geometries (Geom.) across all considered lengths (Len.).r_0_ [nm] specifies the position of the harmonic potential along the reaction coordinate, while κ [kJ/(mol·nm^2^)] indicates the spring constants.(PDF)

S1 Fig Loop-folding free energy profiles for five loop geometries and loop lengths from 1 to 4 nt. Columns correspond to loop geometries, while rows correspond to loop lengths. Profiles for the 1-nt diagonal and 1-nt long-distance propeller loops could not be reliably determined, as explained in the main text. Green and red vertical bands indicate regions of the reaction coordinate (x-axis) corresponding to the folded and unfolded states, respectively (see section A in S1 Text for definitions). Shaded areas around each curve represent the estimated free energy uncertainty. For the convergence of the profiles, see S2–S6 Figs.
(TIF)

S2 Fig Convergence of the loop-folding free energy profiles for short-distance propeller loops of all four lengths. Free energy profiles were evaluated after each 400 ns interval of simulation time, from 400 ns to 4000 ns. Profiles evaluated between 400 ns and 3600 ns are shown as solid lines, while the final profile, evaluated after the full 4000 ns simulation time, is shown as a dashed line.
(TIF)

S3 Fig Convergence of the loop-folding free energy profiles for narrow-groove lateral loops of all four lengths. Free energy profiles were evaluated after each 400 ns interval of simulation time, from 400 ns to 4000 ns. Profiles evaluated between 400 ns and 3600 ns are shown as solid lines, while the final profile, evaluated after the full 4000 ns simulation time, is shown as a dashed line.
(TIF)

S4 Fig Convergence of the loop-folding free energy profiles for wide-groove lateral loops of all four lengths. Free energy profiles were evaluated after each 400 ns interval of simulation time, from 400 ns to 4000 ns. Profiles evaluated between 400 ns and 3600 ns are shown as solid lines, while the final profile, evaluated after the full 4000 ns simulation time, is shown as a dashed line.
(TIF)

S5 Fig Convergence of the loop-folding free energy profiles for diagonal loops of all three lengths. Free energy profiles were evaluated after each 400 ns interval of simulation time, from 400 ns to 4000 ns. Profiles evaluated between 400 ns and 3600 ns are shown as solid lines, while the final profile, evaluated after the full 4000 ns simulation time, is shown as a dashed line.
(TIF)

S6 Fig Convergence of the loop-folding free energy profiles for long-distance propeller loops of all three lengths. Free energy profiles were evaluated after each 400 ns interval of simulation time, from 400 ns to 4000 ns. Profiles evaluated between 400 ns and 3600 ns are shown as solid lines, while the final profile, evaluated after the full 4000 ns simulation time, is shown as a dashed line.
(TIF)

S7 Fig Free energy profiles for binding of unconnected G-tracts. Green and red vertical bands indicate regions of the reaction coordinate (x-axis) corresponding to the bound and unbound states, respectively. Shaded areas around each curve represent the estimated free energy uncertainty. For the convergence of the profiles, see S8 Fig.
(TIF)

S8 Fig Convergence of the G-tract binding free energy profiles for *Anti-Anti* and *Syn-Anti* G-tract conformations. Free energy profiles were evaluated after each 200 ns interval of simulation time, from 200 ns to 2000 ns. Profiles evaluated between 200 ns and 1800 ns are shown as solid lines, while the final profile, evaluated after the full 2000 ns simulation time, is shown as a dashed line.
(TIF)

S9 Fig Comparison of loop-folding free energy profiles calculated with two potential energy models: bsc1 and bsc1-VdW for five loop geometries and loop length 3. Green and red vertical bands indicate regions of the reaction coordinate (x-axis) corresponding to the folded and unfolded states, respectively (see section A in S1 Text for definitions). Shaded areas around each curve represent the estimated free energy uncertainty.
(TIF)

S10 Fig Shortest surface paths between loop attachment points for all five loop geometries in two-tetrad G4s. Two-tetrad G-core structures showing loop attachment points defined by the C3’ (red spheres) and C4’ (green spheres) atoms for all five loop geometries. The shortest surface paths connecting the attachment points are depicted in brown stick representation (see section B in S1 Text for details of path determination). For each geometry, the corresponding distributions of shortest-path lengths, calculated from 1,000 G-core configurations sampled from the MD-generated ensemble, are shown below the structures.
(TIF)

S11 Fig Shortest surface paths between loop attachment points for propeller loop geometries in three-tetrad G4s. Three-tetrad G-core structures showing loop attachment points defined by the C3’ (red spheres) and C4’ (green spheres) atoms for short- and long-distance propeller geometries. The shortest surface paths connecting the attachment points are depicted in brown stick representation (see section B in S1 Text for details of path determination). For both geometries, the corresponding distributions of shortest-path lengths, calculated from 1,000 G-core configurations sampled from the MD-generated ensemble, are shown below the structures.
(TIF)

S12 Fig Free energy profiles for the transition between two-tetrad- and three-tetrad-spanning conformations of short- and long-distance propeller geometries for four loop lengths. For loop lengths of 1 and 2 nt, free energy profiles for long-distance propellers could not be determined, as explained in the main text. In each profile, free energy minima corresponding to the two-tetrad- and three-tetrad-spanning conformations are indicated by circle and diamond markers, respectively. Shaded areas around each curve represent the estimated free energy uncertainty. For the convergence of the profiles, see S13 and S14 Figs.
(TIF)

S13 Fig Convergence of the free energy profiles for the transition between two-tetrad- and three-tetrad-spanning conformations of short-distance propeller loop for four loop lengths. Free energy profiles were evaluated after each 200 ns interval of simulation time, from 200 ns to 2000 ns. Profiles evaluated between 200 ns and 1800 ns are shown as solid lines, while the final profile, evaluated after the full 2000 ns simulation time, is shown as a dashed line.
(TIF)

S14 Fig Convergence of the free energy profiles for the transition between two-tetrad- and three-tetrad-spanning conformations of long-distance propeller loop for two loop lengths. Free energy profiles were evaluated after each 200 ns interval of simulation time, from 200 ns to 2000 ns. Profiles evaluated between 200 ns and 1800 ns are shown as solid lines, while the final profile, evaluated after the full 2000 ns simulation time, is shown as a dashed line.
(TIF)

S15 Fig Depiction of attachment points positions in propeller loops spanning two vs. three tetrads. Scheme showing two positions of the 3^′^-end loop attachment point (C3’ atom; green sphere) within the three-tetrad G-core scaffold: i) when the propeller loop spans two-tetrads and ii) when the propeller loop spans three-tetrads, in case of long-distance (left) and short-distance (right) propellers. Arrows show how the position of the C3’ atom changes relatively to the 5^′^-end attachment point (C4’ atom; red sphere) upon the change of the propeller loop’s span from two- to three-tetrad. Specifically, magenta arrows show displacement of C3’ atom in a direction that moves it apart from the C4’ atom, while yellow arrow shows displacement of C3’ atom in direction bringing it closer to the C4’ atom.
(TIF)

S16 Fig All 26 standard G4 topologies. G-tracts are depicted as gray rods, and loops are color-coded according to their geometry: yellow – short-distance propeller; red – long-distance propeller; cyan – narrow-groove lateral; dark blue – wide-groove lateral; magenta – diagonal. The standard designation of each topology is shown below the corresponding scheme. In this notation, *p*, *l*, and *d* denote propeller, lateral, and diagonal loops, respectively, while “-” and “ + ” indicate anticlockwise and clockwise loop progression from the 5^′^-end to the 3^′^-end. The geometries of the three loops in each topology are given in brackets.
(TIF)

S17 FigHeatmaps of loop-folding free energies with substituted missing values for two- and three-tetrad G4s.**Heatm**aps show loop-folding free energies ($\Delta G_{fold}$) for five loop geometries (x-axis) across four loop lengths (y-axis) in two-tetrad (**A**) and three-tetrad (**B**) G4s. Entries marked with an asterisk denote loop geometries for which $\Delta G_{fold}$ could not be determined and were therefore substituted with the value corresponding to the shortest loop of the same geometry for which $\Delta G_{fold}$ was successfully calculated. The thick dashed line separates loop geometries observed in experimental G4 structures (left) from those not observed experimentally (right).(TIF)

S18 FigDetailed additive folding free energies ($\Delta G_{add}$) for 8 out of 15 experimental sequences forming three-tetrad G4s.Bar plots show additive folding free energies ($\Delta G_{add}$; x-axis) for all 26 G4 topologies (y-axis) across 15 sequences forming three-tetrad G-quadruplexes (8 sequences shown; see S19 Fig for the remaining 7). Sequences are labeled above each plot using three-digit codes denoting loop lengths. For each sequence, topologies are ordered from most to least stable according to $\Delta G_{add}$. Topologies observed experimentally for a given sequence are indicated by black outlines.(TIF)

S19 FigDetailed additive folding free energies ($\Delta G_{add}$) for 7 out of 15 experimental sequences forming three-tetrad G4s.Bar plots show additive folding free energies $\Delta G_{add}$ (x-axis) calculated for all 26 topologies (y-axis) across 15 sequences forming three-tetrad G-quadruplexes (7 sequences are shown; for the remaining 8 see S18 Fig). Sequences are labeled above each plot using three-digit codes denoting loop lengths. For each sequence, topologies are ordered from most to least stable according to $\Delta G_{add}$. Topologies observed experimentally for a given sequence are indicated by black outlines.(TIF)

S20 FigDetailed additive folding free energies ($\Delta G_{add}$) for 8 experimental sequences forming two-tetrad G4s.Bar plots show additive folding free energies $\Delta G_{add}$ (x-axis) calculated for all 26 topologies (y-axis) across 8 sequences forming two-tetrad G-quadruplexes. Sequences are labeled above each plot using three-digit codes denoting loop lengths. For each sequence, topologies are ordered from most to least stable according to $\Delta G_{add}$. Topologies observed experimentally for a given sequence are indicated by black outlines.(TIF)

S21 Fig Distributions of loop attachment-point separations in two-tetrad G4s. Distributions calculated as the shortest surface path along the G-core, are shown for all five loop geometries (columns) and loop lengths from 1 to 4 nt (rows). For comparison, distributions of relaxed, geometry-specific attachment-point separations calculated for the loop-free two-tetrad G-core are shown in light brown. Mean values of each distribution are indicated by dashed vertical lines. Separation mismatch values for each loop geometry and length, defined as the difference between the mean relaxed separation and the mean loop-enforced separation, are reported in the upper part of each subplot.
(TIF)

S22 Fig Distributions of loop attachment-point separations in three-tetrad G4s. Distributions calculated as the shortest surface path along the G-core, are shown for all five loop geometries (columns) and loop lengths from 1 to 4 nt (rows). For comparison, distributions of relaxed, geometry-specific attachment-point separations calculated for the loop-free two-tetrad G-core are shown in light brown. Mean values of each distribution are indicated by dashed vertical lines. Separation mismatch values for each loop geometry and length, defined as the difference between the mean relaxed separation and the mean loop-enforced separation, are reported in the upper part of each subplot.
(TIF)
