# Supplementary material for: Can intrinsic loop energetics predict G-Quadruplex topology?
Source: PLoS Comput Biol. 2026 Aug 18;22(8):e1014542. doi: 10.1371/journal.pcbi.1014542 (PMC13497301; doi:10.1371/journal.pcbi.1014542)
Supplement: Supplementary file 1 [file pcbi.1014542.s001.pdf]

Supporting Information for: Can Intrinsic Loop Energetics Predict G-Quadruplex Topology?

Michał Jurkowski,[†] Mateusz Kogut,[*,†] Mikołaj Ławicki,[†] and Jacek Czub[*,†,‡]

†*Department of Physical Chemistry, Gdańsk University of Technology, Narutowicza St 11/12, 80-233 Gdańsk, Poland*

‡*BioTechMed Center, Gdańsk University of Technology, Narutowicza St 11/12, 80-233, Gdańsk, Poland*

E-mail: mateusz.kogut@pg.edu.pl; jacek.czub@pg.edu.pl

Phone: +48 58 347 27 69; +48 58 347 16 10

A: Derivation of folding free energies from free energy profiles

To derive loop-folding free energies from the free energy profiles, we calculated the difference between the folded- and unfolded-state free energies. The folded state was defined as the region of the reaction coordinate between 0.28 and 0.31 nm, corresponding to configurations with eight fully formed hydrogen bonds between the 3'-end G-tract and the preassembled core. The folded-state free energy was taken as the minimum free energy within this region. Because fully unfolded states – characterized by fully extended loops – map to different regions of the reaction coordinate depending on loop length, the unfolded state was defined separately for each loop length. Specifically, for a given loop length, the unfolded state was identified as the tail region of the reaction coordinate over which all free energy profiles ex-

hibit a strong linear dependence, as quantified by a Pearson correlation coefficient exceeding 0.99. This highly linear increase in free energy is attributed to the intrinsic cost of loop extension, with minimal contribution from interactions with the G4 core, and therefore serves as a suitable definition of the unfolded state. The unfolded-state free energy was taken as the value at the midpoint of this reaction-coordinate interval.

B: Separation between loop attachment points – shortest path calculations

The separation distance between loop attachment points was defined as the shortest path along the surface of the guanine core, between the C3' and C4' atoms of the guanosines connected by the loop. Distances were calculated separately for G-cores in the absence and in the presence of a folded loop, yielding relaxed and loop-enforced separation distances, respectively.

Relaxed separation distances were computed using structures obtained from replica-exchange umbrella sampling (REUS) simulations of G-tract binding (REUS window 1; see Table S3). For each of the *anti-anti* and *syn–anti* G-core conformations, 1,000 bound-state configurations of a fully formed G-core were randomly selected.

For each configuration, a Connolly surface was generated using the GROMACS SASA tool with a probe radius of 0.18 nm, corresponding to the radius of a DNA phosphate group and approximating the effective thickness of the loop backbone. The resulting surface grids represent regions of the G-core surface accessible to the loop and had an average density of approximately 70 points per nm^2.

Surface grids were converted into graphs by treating each grid point as a node. Edges were introduced between nodes separated by less than 0.3 nm, and edge weights were assigned as Euclidean distances between connected nodes. Shortest paths between the loop attachment points were computed using Dijkstra's algorithm. Short- and long-distance propeller loop

geometries were evaluated on the surface of the anti–anti G-core, while narrow- and wide-groove lateral and diagonal loop geometries were evaluated on the surface of the syn–anti G-core. Path lengths were calculated as the sum of the edge weights along the shortest path.

Loop-enforced separation distances were computed using structures obtained from REUS simulations of loop folding for all considered loop geometries and loop lengths. From each simulation, 1,000 configurations corresponding to the folded state (REUS window 1; see S2 Table) were selected. For each configuration, the G-core structure was extracted, and a Connolly surface was generated using the same parameters as for the relaxed G-core. The resulting surface grids were converted into graphs, and shortest paths between loop attachment points were determined using the Dijkstra's algorithm as described above.

Relaxed loop separation distances for short- and long-distance propeller loops in three-tetrad G4s were calculated using structures obtained from an additional 500-ns molecular dynamics (MD) simulation of the three-tetrad G-core. The initial structure was identical to that used for the free-energy calculations of the propeller loop transition between two- and three-tetrad-spanning conformations, except that no G-tract truncation was applied. System preparation followed the procedure described in 'Preparation of simulation systems' in the main text. The integrity of the G-core was maintained by restraining all 16 Hoogsteen hydrogen bonds between the four G-tracts using a harmonic potential with a spring constant of 500 $kJ/(mol{\cdot}nm^2)$. Two K^+ ions were restrained at the centers of mass of the O6 atoms in the lower–middle and middle–upper tetrads using harmonic potentials with the same spring constant (500 $kJ/(mol{\cdot}nm^2)$).

From the resulting MD trajectory, 1,000 G-core configurations were selected and processed using the same shortest-path calculation workflow described above to compute the separation distances between attachment points for short- and long-distance propeller loops.

Loop-enforced separation distances for short- and long-distance propeller loops in three-tetrad G4s were calculated using G-core structures extracted from REUS simulations of the conformational transition between two-tetrad- and three-tetrad-spanning states of the

propeller loops. For each geometry, 1,000 configurations were selected from the three-tetrad-spanning state (REUS window 8; see S4 Table). In the case of long-distance propeller loops, the vacancy created by truncation of one G-tract was filled with a guanine base prior to Connolly surface generation to prevent artificial shortening of surface paths through the vacancy. Shortest paths between loop attachment points were then calculated following the procedure described above.
