# Supplementary material for: Can intrinsic loop energetics predict G-Quadruplex topology?
Source: PLoS Comput Biol. 2026 Aug 18;22(8):e1014542. doi: 10.1371/journal.pcbi.1014542 (PMC13497301; doi:10.1371/journal.pcbi.1014542)
Supplement: Supplementary file 2 [file pcbi.1014542.s002.pdf]

Single-loop system			Parent G4 structure		
G-core	Loop geometry	Loop length	Sequence	Topology	Glycosidic bond angles
two-tetrad	*sdp*	1	111	−p−p−p	*anti-anti*
		2	222	−p−p−p	*anti-anti*
		3	333	−p−p−p	*anti-anti*
		4	444	−p−p−p	*anti-anti*
	ldp	2	213	+p+l+p	*anti-anti*
		3	312	+p+l+p	*anti-anti*
		4	433	+p+l+p	*anti-anti*
	ngl	1	122	+l+l+l	*syn-anti*
		2	222	+l+l+l	*syn-anti*
		3	323	+l+l+l	*syn-anti*
		4	444	+l+l+l	*syn-anti*
	wgl	1	122	−l−l−p	*syn-anti*
		2	222	−l−l−p	*syn-anti*
		3	322	−l−l−p	*syn-anti*
		4	444	−l−l−p	*syn-anti*
	d	2	244	d+pd	*syn-anti*
		3	334	d+pd	*syn-anti*
		4	444	d+pd	*syn-anti*
three-tetrad	*sdp*	1	111	−p−p−p	*anti-anti-anti*
		2	211	−p−p−p	*anti-anti-anti*
		3	311	−p−p−p	*anti-anti-anti*
		4	411	−p−p−p	*anti-anti-anti*
	ldp	3	341	+p+l+p	*anti-anti-anti*
		4	441	+p+l+p	*anti-anti-anti*
