# Supplementary material for: Can intrinsic loop energetics predict G-Quadruplex topology?
Source: PLoS Comput Biol. 2026 Aug 18;22(8):e1014542. doi: 10.1371/journal.pcbi.1014542 (PMC13497301; doi:10.1371/journal.pcbi.1014542)
Supplement: Supplementary file 3 [file pcbi.1014542.s003.pdf]

Sim. system			REUS window																	
Geom.	Len.		1	2	3	4	5	6	7	8	9	10	11	12	13	14	15	16	17	18
sdp	1	d_0	0.30	0.38	0.45	0.52	0.60	0.75	0.90	1.05	1.30	1.55	1.80	2.05	–	–	–	–	–	–
		κ	250.0	750.0	750.0	750.0	250.0	250.0	250.0	250.0	250.0	250.0	250.0	250.0	–	–	–	–	–	–
	2	d_0	0.29	0.38	0.45	0.52	0.60	0.68	0.75	0.90	1.05	1.30	1.55	1.80	2.05	2.30	–	–	–	–
		κ	250.0	750.0	750.0	750.0	750.0	750.0	250.0	250.0	250.0	250.0	250.0	250.0	250.0	250.0	–	–	–	–
	3	d_0	0.29	0.38	0.45	0.52	0.60	0.70	0.75	0.90	1.05	1.30	1.55	1.80	2.05	2.30	2.55	2.80	–	–
		κ	250.0	750.0	750.0	750.0	250.0	750.0	250.0	250.0	250.0	250.0	250.0	250.0	250.0	250.0	250.0	250.0	–	–
	4	d_0	0.29	0.38	0.45	0.52	0.60	0.68	0.75	0.90	1.05	1.30	1.55	1.80	2.05	2.30	2.55	2.80	3.05	3.30
		κ	250.0	750.0	750.0	750.0	750.0	750.0	750.0	250.0	250.0	250.0	250.0	250.0	250.0	250.0	250.0	250.0	250.0	250.0
ldp	2	d_0	0.29	0.38	0.45	0.52	0.60	0.68	0.75	0.90	1.05	1.30	1.55	1.80	2.05	2.30	–	–	–	–
		κ	1500.0	1250.0	1000.0	750.0	750.0	750.0	500.0	250.0	250.0	250.0	250.0	250.0	250.0	250.0	–	–	–	–
	3	d_0	0.29	0.38	0.45	0.52	0.60	0.68	0.75	0.90	1.05	1.30	1.55	1.80	2.05	2.30	2.55	2.80	–	–
		κ	250.0	1250.0	1000.0	750.0	750.0	500.0	250.0	250.0	250.0	250.0	250.0	250.0	250.0	250.0	250.0	250.0	–	–
	4	d_0	0.29	0.38	0.45	0.52	0.60	0.68	0.75	0.90	1.05	1.30	1.55	1.80	2.05	2.30	2.55	2.80	3.05	3.30
		κ	250.0	1250.0	750.0	750.0	750.0	500.0	250.0	250.0	250.0	250.0	250.0	250.0	250.0	250.0	250.0	250.0	250.0	250.0
ngl	1	d_0	0.30	0.38	0.45	0.52	0.60	0.75	0.90	1.05	1.30	1.55	1.80	2.05	–	–	–	–	–	–
		κ	250.0	1250.0	750.0	750.0	500.0	250.0	250.0	250.0	250.0	250.0	250.0	250.0	–	–	–	–	–	–
	2	d_0	0.29	0.38	0.45	0.52	0.60	0.68	0.75	0.90	1.05	1.30	1.55	1.80	2.05	2.30	–	–	–	–
		κ	250.0	1250.0	750.0	750.0	750.0	500.0	250.0	250.0	250.0	250.0	250.0	250.0	250.0	250.0	–	–	–	–
	3	d_0	0.29	0.38	0.45	0.52	0.60	0.68	0.75	0.90	1.05	1.30	1.55	1.80	2.05	2.30	2.55	2.80	–	–
		κ	250.0	1250.0	750.0	750.0	750.0	500.0	250.0	250.0	250.0	250.0	250.0	250.0	250.0	250.0	250.0	250.0	–	–
	4	d_0	0.29	0.38	0.45	0.52	0.60	0.68	0.75	0.90	1.05	1.30	1.55	1.80	2.05	2.30	2.55	2.80	3.05	3.30
		κ	250.0	1250.0	750.0	750.0	750.0	500.0	250.0	250.0	250.0	250.0	250.0	250.0	250.0	250.0	250.0	250.0	250.0	250.0
wgl	1	d_0	0.30	0.38	0.45	0.52	0.60	0.75	0.90	1.05	1.30	1.55	1.80	2.05	–	–	–	–	–	–
		κ	250.0	750.0	750.0	750.0	250.0	250.0	250.0	250.0	250.0	250.0	250.0	250.0	–	–	–	–	–	–
	2	d_0	0.29	0.38	0.45	0.52	0.60	0.68	0.75	0.90	1.05	1.30	1.55	1.80	2.05	2.30	–	–	–	–
		κ	250.0	750.0	750.0	750.0	750.0	750.0	250.0	250.0	250.0	250.0	250.0	250.0	250.0	250.0	–	–	–	–
	3	d_0	0.29	0.38	0.45	0.52	0.60	0.68	0.75	0.90	1.05	1.30	1.55	1.80	2.05	2.30	2.55	2.80	–	–
		κ	250.0	750.0	750.0	750.0	750.0	750.0	250.0	250.0	250.0	250.0	250.0	250.0	250.0	250.0	250.0	250.0	–	–
	4	d_0	0.29	0.38	0.45	0.52	0.60	0.68	0.75	0.90	1.05	1.30	1.55	1.80	2.05	2.30	2.55	2.80	3.05	3.30
		κ	250.0	750.0	750.0	750.0	750.0	750.0	250.0	250.0	250.0	250.0	250.0	250.0	250.0	250.0	250.0	250.0	250.0	250.0
d	2	d_0	0.29	0.38	0.45	0.52	0.60	0.68	0.75	0.90	1.05	1.30	1.55	1.80	2.05	2.30	–	–	–	–
		κ	250.0	1250.0	750.0	750.0	750.0	500.0	250.0	250.0	250.0	250.0	250.0	250.0	250.0	250.0	–	–	–	–
	3	d_0	0.29	0.38	0.45	0.52	0.60	0.68	0.75	0.90	1.05	1.30	1.55	1.80	2.05	2.30	2.55	2.80	–	–
		κ	250.0	1250.0	750.0	750.0	750.0	500.0	250.0	250.0	250.0	250.0	250.0	250.0	250.0	250.0	250.0	250.0	–	–
	4	d_0	0.29	0.38	0.45	0.52	0.60	0.68	0.75	0.90	1.05	1.30	1.55	1.80	2.05	2.30	2.55	2.80	3.05	3.30
		κ	250.0	1250.0	750.0	750.0	750.0	500.0	250.0	250.0	250.0	250.0	250.0	250.0	250.0	250.0	250.0	250.0	250.0	250.0
