# Supplementary material for: Can intrinsic loop energetics predict G-Quadruplex topology?
Source: PLoS Comput Biol. 2026 Aug 18;22(8):e1014542. doi: 10.1371/journal.pcbi.1014542 (PMC13497301; doi:10.1371/journal.pcbi.1014542)
Supplement: Supplementary file 4 [file pcbi.1014542.s004.pdf]

Sim. system		REUS window													
Glycosidic bond conformation		1	2	3	4	5	6	7	8	9	10	11	12	13	14
anti-anti	d_0	0.29	0.38	0.45	0.52	0.60	0.68	0.75	0.90	1.05	1.30	1.55	1.80	2.05	2.30
	κ	250.0	1250.0	1000.0	750.0	750.0	750.0	250.0	250.0	250.0	250.0	250.0	250.0	250.0	250.0
syn-anti	d_0	0.29	0.38	0.45	0.52	0.60	0.68	0.75	0.90	1.05	1.30	1.55	1.80	2.05	2.30
	κ	250.0	2500.0	2000.0	1500.0	1000.0	750.0	250.0	250.0	250.0	250.0	250.0	250.0	250.0	250.0
