# Supplementary material for: Can intrinsic loop energetics predict G-Quadruplex topology?
Source: PLoS Comput Biol. 2026 Aug 18;22(8):e1014542. doi: 10.1371/journal.pcbi.1014542 (PMC13497301; doi:10.1371/journal.pcbi.1014542)
Supplement: Supplementary file 5 [file pcbi.1014542.s005.pdf]

| Sim. system | | | REUS window | | | | | | | |
|---|---|---|---|---|---|---|---|---|---|---|
| Geom. | Len. | | 1 | 2 | 3 | 4 | 5 | 6 | 7 | 8 |
| *sdp* | 1 | $d_0$ | -0.20 | -0.14 | -0.08 | -0.02 | 0.02 | 0.08 | 0.14 | 0.20 |
| | | $\kappa$ | 500.0 | 750.0 | 1250.0 | 1750.0 | 1750.0 | 1250.0 | 750.0 | 500.0 |
| | 2 | $d_0$ | -0.20 | -0.14 | -0.08 | -0.02 | 0.02 | 0.08 | 0.14 | 0.20 |
| | | $\kappa$ | 500.0 | 750.0 | 1250.0 | 1750.0 | 1750.0 | 1250.0 | 750.0 | 500.0 |
| | 3 | $d_0$ | -0.20 | -0.14 | -0.08 | -0.02 | 0.02 | 0.08 | 0.14 | 0.20 |
| | | $\kappa$ | 500.0 | 750.0 | 1250.0 | 1750.0 | 1750.0 | 1250.0 | 750.0 | 500.0 |
| | 4 | $d_0$ | -0.20 | -0.14 | -0.08 | -0.02 | 0.02 | 0.08 | 0.14 | 0.20 |
| | | $\kappa$ | 500.0 | 750.0 | 1250.0 | 1750.0 | 1750.0 | 1250.0 | 750.0 | 500.0 |
| *ldp* | 3 | $d_0$ | -0.20 | -0.14 | -0.08 | -0.02 | 0.02 | 0.08 | 0.14 | 0.20 |
| | | $\kappa$ | 500.0 | 750.0 | 1250.0 | 1750.0 | 1750.0 | 1250.0 | 1250.0 | 1250.0 |
| | 4 | $d_0$ | -0.20 | -0.14 | -0.08 | -0.02 | 0.02 | 0.08 | 0.14 | 0.20 |
| | | $\kappa$ | 500.0 | 750.0 | 1250.0 | 1750.0 | 1750.0 | 1250.0 | 1250.0 | 1250.0 |
